# Phytochemical Content and Antioxidant Activities of *Ficus carica* Extracts

**DOI:** 10.1155/ianc/3209992

**Published:** 2026-04-22

**Authors:** Sadia Zulfiqar, Masood Sadiq Butt, Aqsa Chatha, Maryam S. Hafiz

**Affiliations:** ^1^ National Institute of Food Science and Technology, University of Agriculture, Faisalabad, Pakistan, uam.edu.ng; ^2^ Department of Food Science and Human Nutrition, University of Illinois, Urbana-Champaign, Illinois, USA, illinois.edu; ^3^ Department of Clinical Nutrition, Faculty of Applied Medical Sciences, King Abdulaziz University, Jeddah, Saudi Arabia, kau.edu.sa

**Keywords:** antioxidant activity, *Ficus carica*, fig, flavonoids, functional foods, phenolic compounds

## Abstract

Fig (*Ficus carica*) is a fruit rich in bioactive compounds with potential health benefits, including the ability to modulate blood glucose levels and reduce oxidative stress. Efficient extraction of these bioactive molecules is essential to enhance their applications in functional and nutraceutical formulations. The efficiency of recovery, however, depends strongly on solvent polarity and extraction parameters. This study evaluated the effect of solvent polarity and extraction time on phytochemical recovery and antioxidant activity of fig powder. The phytochemical evaluation revealed significant variation among solvents. Methanolic extracts showed the highest total polyphenol content (3.15 ± 0.1 mg GAE/g) and exhibited superior antioxidant activities compared to aqueous and ethanolic extracts, particularly at 45 min of extraction. Antioxidant potential was confirmed by DPPH radical scavenging activity (70.2 ± 2.3%), ferric reducing antioxidant power (FRAP) (16.6 ± 0.8 μM·TE/g), ABTS radical cation inhibition (9.5 ± 0.4 μM·TE/g), metal chelating activity (67 ± 3.8%), and *β*‐carotene and linoleic acid assay (66.53 ± 3.68%). Proximate analysis confirmed fig powder as a fiber‐rich, low‐fat matrix suitable for functional food applications. These findings indicate that solvent type and extraction time directly influence bioactive yield and antioxidant performance. Methanolic extraction at 45 min was determined to be the optimal condition for maximizing phenolic recovery and antioxidant activity. The results reinforce *Ficus carica* as a valuable natural source of phenolics and antioxidants with promising applications in the formulation of functional foods and nutraceuticals.

## 1. Introduction

*Ficus carica*, commonly known as fig, is a fruit of the Moraceae family that is widely cultivated in tropical and subtropical regions of the Mediterranean region, holding a unique place in both traditional diets and modern nutritional research [1–3]. They have been cultivated for thousands of years not only for their distinctive sweetness and culinary versatility but also for their rich nutritional profile and therapeutic potential. Figs are considered a significant source of dietary carbohydrates, essential vitamins (A, B1, B2, B6, C, and K), and minerals such as potassium, calcium, magnesium, phosphorus, and iron, which play vital roles in maintaining human health and preventing nutrient deficiencies [4, 5]. The exceptional balance between macronutrients and bioactive compounds makes figs an ideal candidate for development into functional food products that promote well‐being and mitigate disease risk [4].

Figs are recognized for their abundance of phytochemicals, including phenolic acids, flavonoids, anthocyanins, tannins, and organic acids, which contribute significantly to their antioxidant and health‐protective properties [6]. Figs contain diverse phenolic acids and flavonoids, including caffeoylquinic acids, quercetin derivatives, ferulic acid, and catechins, which collectively contribute to their antioxidant capacity [7]. These secondary metabolites act as free radical scavengers, metal chelators, and reducing agents, mechanisms that collectively help to prevent oxidative damage at the cellular level. Recent studies illustrate that the bioactive moieties present in fig fruit are of nutraceutical value [8–11].

Processing fresh figs into dehydrated forms such as powders extends their shelf life while concentrating their nutrients and phytochemicals [5]. Fig powder offers an efficient, stable, and versatile form suitable for incorporation into cereals, bakery items, nutraceuticals, and meal replacement formulas. Moreover, compared to fresh fruit, fig powder exhibits improved storage stability and can serve as a natural sweetener, fiber enhancer, and antioxidant additive in various food products. Previous reports also suggest that fig‐based products, besides their free radical scavenging ability, may exert additional health‐promoting properties, including improved gut health, glycemic regulation, and lipid homeostasis [12, 13]. Studies have demonstrated that processing conditions such as temperature, dehydration technique, and particle size can significantly influence the retention of active compounds in fruit‐derived powders [14]. When appropriately processed, fig powder retains high levels of natural sugars (glucose and fructose), fiber, phenolic compounds, and minerals while exhibiting strong antioxidant potential. These properties make fig powder an appealing candidate for functional food development and replacing refined sugars in formulations intended for health‐conscious or diabetic populations [5].

Given these nutritional and biochemical attributes, the current study aims to investigate the effect of solvent type and extraction time on polyphenol and flavonoid recovery from fig powder and to evaluate antioxidant activity using multiple in vitro assays.

## 2. Materials and Methods

The research was conducted in the Functional and Nutraceutical Food Research Section, National Institute of Food Science and Technology, University of Agriculture, Faisalabad, Pakistan. The fig fruit was purchased fresh from the local market. They were washed, oven‐dried at 50°C, and ground (Renker, Model: GMO 1 grinder) for further analysis. Reagents and standards were purchased from Merck (Merck KGaA, Darmstadt, Germany) and Sigma‐Aldrich (Sigma‐Aldrich Tokyo, Japan).

### 2.1. Proximate Analysis

#### 2.1.1. Moisture Content

Moisture content of dried fig fruit was determined by drying the sample in an Air Forced Draft Oven (Model: DO‐1‐30/02, PCSIR, Pakistan) at 105 ± 5°C till constant weight according to the AOAC (2023) Method No. 934‐01 [15].

#### 2.1.2. Crude Protein

Protein content of the fig sample was determined using the Kjeltec Apparatus (Model: D‐40599, Behr Labor‐Technik GmbH, Germany) as per the procedure described in AOAC (2023) [15]. In brief, dried figs were digested with conc. H_2_SO_4_ by using a digestion mixture (K_2_SO_4_:FeSO_4_:CuSO_4_ as 100:5:10) until the color was transparent greenish. The digested material was then diluted up to 250 mL in a volumetric flask. 10 mL of 40% NaOH with 10 mL of the digested sample was taken in a distillation apparatus, whereas liberated ammonia was collected in a separate beaker containing 4% boric acid solution, using methyl red as an indicator. Consequently, ammonium borate was formed, which was used for nitrogen determination in the sample. Thus, the percentage of nitrogen in the sample was estimated by titrating the distillate against a 0.1 N H_2_SO_4_ solution till light golden coloration. Crude protein content was calculated by multiplying nitrogen percent (N %) by a factor of 6.25.

(1) %=Vol. of 0.10.0014 N H2SO4××Vol. of dilution 250 mLVol. of distillate taken×weight of sample×100 N,

(2) Crude protein %=nitrogen %×6.25.

#### 2.1.3. Crude Fat

Crude fat content was determined using hexane as solvent in the Soxtec System (Model: H‐2 1045 Extraction Unit, Hoganas, Sweden) following the protocol of AOAC (2021) [15]. In brief, 5 g of the homogenized sample was dried to constant weight at 100°C and placed in a cellulose extraction thimble. Extraction was carried out with hexane for 6 h in a Soxhlet apparatus. After extraction, the solvent was evaporated using a rotary evaporator, and the fat residue was dried at 102°C until a constant mass was reached. Results were expressed as a percentage of the original sample weight.

#### 2.1.4. Crude Fiber

Crude fiber in fat‐free samples was estimated by digesting first with 1.25% H_2_SO_4_ for 30 min and then with 1.25% NaOH solution through Labconco Fibertech (Labconco Corporation, Kansas, USA) as described in AOAC (2021) [15]. Afterward, the sample was filtered and washed with distilled water. The residue was weighed and placed in a muffle furnace at a temperature of 550°C–650°C till gray or white ash was obtained. The crude fiber percentage was estimated according to the following expression:

(3) crude fiber %=weight loss on ignition gweight of the sample g×100.

#### 2.1.5. Total Ash

Ash in each dry sample was determined by direct incineration in a muffle furnace (MF‐1/02, PCSIR, Pakistan) at 550°C–600°C after charring, till grayish white residue (AOAC, 2021) [15].

#### 2.1.6. Nitrogen‐Free Extract (NFE)

NFE in dried fig samples was calculated according to the given expression

(4) NFE%=100−CP%+CF12%+CF%+Ash%,

where CP = crude protein, CF1 = crude fat, and CF2 = crude fiber.

### 2.2. Extraction of Polyphenols

Extracts were prepared using water and aqueous methanol (50% v/v) and aqueous ethanol (50% v/v), following the methods described by Caliskan and Polat (2011) with slight modifications [16]. Methanol was used as a reference solvent due to its high efficiency for phenolic extraction in analytical studies. Extraction treatments involved three time intervals (30, 45, and 60 min) at a constant temperature of 50°C. In addition, solvent extracts (aqueous methanol and aqueous ethanol), excluding water extracts, were filtered and then concentrated using a rotary evaporator (Eyela, Japan).

### 2.3. Phytochemical Screening Tests

#### 2.3.1. Total Phenolic Contents (TPCs)

TPC in fig fruit was estimated using the Folin–Ciocalteu method as mentioned by Caliskan and Polat (2011) [17]. For this purpose, 50 μL of each extract was added to the test tube containing 250 μL of 10% Folin–Ciocalteu’s reagent and 750 μL of 20% sodium carbonate solution, and the final volume was made up to 5 mL with distilled water. After 2 hours, absorbance was measured at 765 nm using a UV–visible light spectrophotometer (CECIL CE7200) against a control having all reaction reagents except the sample extract. Gallic acid (25, 50, 100, 125, 250, and 500 μg/mL) was used as a standard to measure total polyphenols in fig extracts.

Total polyphenols were expressed as gallic acid equivalent (GAE) (mg gallic acid/g of dry weight) using the following formula:

(5) C=c×Vm,

where *C* = TPC (mg/g plant extract, in GAE), *c* = concentration of gallic acid (mg/mL), *V* = volume of the extract (mL), and *M* = weight of the fig fruit (g).

#### 2.3.2. Flavonoids

Flavonoid contents were determined using a spectrophotometric method as described previously. The principle is based on the development of a flavonoid–aluminum complex (pink colored mixture), and catechin (50, 100, 150, 200, 250, and 300 mg/L) was used as a standard to measure total flavonoids in fig extracts. For this purpose, 1 mL of extract, standard, or blank was added to a 10‐mL volumetric flask containing 4 mL of distilled water, followed by the addition of 0.3 mL of 5% sodium nitrite (NaNO_2_). After 5 min, 0.3 mL of 10% AlCl_3_ was added and incubated for 6 min, followed by the addition of 2 mL of 1 M NaOH, and the volume was adjusted to 10 mL with distilled water. Absorbances were measured immediately at 510 nm using a UV–visible light spectrophotometer, and the results were expressed as mg catechin equivalents (CEs) per gram of dry weight of fig.

### 2.4. In Vitro Antioxidant Activity

#### 2.4.1. Free Radical Scavenging Ability (2,2‐Diphenyl‐1‐picrylhydrazyl [DPPH] Assay)

The antioxidant potential of fig extracts for scavenging the radical DPPH was determined using the method previously described with slight modification [18, 19]. In brief, 50 μL of the extract was added to 1950 μL of 60 μM DPPH solution in ethanol. The mixture was incubated in the dark at room temperature for 15 min. Absorbance was measured at 515 nm against the control (extraction solvent + DPPH reagent). Free radical scavenging activity was measured by using the following formula:

(6) radical scavenging activity %=Ac−AsAc×100,

(7) Ac=Absorbance of control As=Absorbance of sample.

#### 2.4.2. *β*‐Carotene and Linoleic Acid Assay (Antioxidant Activity)

Antioxidant activity of the extracts was evaluated based on the coupled oxidation of *β*‐carotene and linoleic acid. The method followed was as described by Zargar et al. [20]. 2 mg of *β*‐carotene was dissolved in 20 mL of chloroform. 3 mL of the aliquot was then dissolved with 40 mg linoleic acid and 400 mg Tween 20. After that, distilled water (100 mL) was added to the *β*‐carotene emulsion and well mixed by a vortex mixer. Aliquots of 3 mL *β*‐carotene emulsion and 0.12 mL phenolic extracts were taken in capped culture tubes and were thoroughly mixed. The capped culture tubes were instantly immersed in a water bath and incubated at 50°C. Oxidation of *β*‐carotene emulsion was estimated after a stay of 30 min by measuring its absorbance through a spectrophotometer at 470 nm. The degradation rate of the extracts was also calculated according to the first‐order kinetic reaction using the following equation:

(8) Inab×1t=sample degradation rate,

where ln = natural log, *a* = initial absorbance on 470 nm at time zero, *b* = absorbance at 470 nm after 30 min, and *t* = time in min.

The antioxidant activity was expressed as percentage inhibition (%) relative to the control.

#### 2.4.3. Ferric Reducing Antioxidant Power (FRAP) Assay

The ferric reducing capacity of fruit extracts was estimated using the method of Gorjanović et al. with some modifications [21]. In brief, the FRAP reagent was prepared by mixing 300 mM acetate buffer (pH 3.6), 5 mM 2, 4, 6‐tripyridyl‐S‐triazine (TPTZ) solution, and FeCl_3_ in the ratio of 10:1:1 (v/v/v). Reducing capability was measured via mixing 50 μL of the sample (1 mg/mL, dissolved in 5% DMSO) with 950 μL of FRAP reagent. The absorbance was recorded at 593 nm after incubation at 37°C for 15 min. Aqueous solutions of FeSO_4_.7 H_2_O (100–1000 μM) were used for calibration, and values were expressed as mmoles per liter Fe (II).

#### 2.4.4. ABTS Assay

The antioxidant activity of fig samples was measured using the ABTS radical scavenging (TEAC) assay as described in previous literature with slight modifications [22]. For this purpose, the ABTS radical stock solution was prepared by mixing 14 mM ABTS stock solution with 4.9 mM potassium peroxodisulfate and then incubating it for 24 h in the dark. The ABTS radical working solution was obtained through diluting the stock solution to reach an initial absorbance of 0.700 ± 0.020 at 734 nm. ABTS radical scavenging activity was tested by adding 50 μL of the sample (1 mg/mL, dissolved in distilled water) to 950 μL of the ABTS radical working solution. The absorbance was taken after 6 min at 734 nm using a UV–visible spectrophotometer (Shimadzu UV‐160A, Kyoto, Japan). Trolox was applied as a standard compound, and the antioxidant capability of the samples was expressed as μM Trolox equivalents (TEs)/g.

### 2.5. Statistical Analysis

Statistical analysis was conducted to determine the level of significance and to compare group means. Significant differences were considered at a *p* value < 0.05. All experiments were carried out in triplicate, and data are presented as mean ± standard deviation (SD). Multiple comparisons between groups were performed using the ANOVA and Bonferroni post hoc test. Statistical analyses were conducted using Microsoft Excel 2021 and IBM SPSS Statistics, Version 31.0.

## 3. Results and Discussion

### 3.1. Proximate Analysis of Fig Powder

Compositional analysis of raw materials is important for evaluating the efficacy of functional or nutraceutical foods. The chemical composition of figs was analyzed and is presented in Table 1. The results revealed that figs contained 80.16 ± 0.24% moisture, 1.24 ± 0.03% crude protein, 1.27 ± 0.03% crude fat, 9.86 ± 0.29% crude fiber, and 0.69 ± 0.02% ash. Furthermore, the NFE of the fig sample was calculated to be 6.78 ± 0.20%, accounting for soluble carbohydrates, suggesting potential utility in functional food applications where low fat, moderate protein, and high fiber are desirable for metabolic health. These results align with established nutritional reports on *Ficus carica*, which emphasize its value as a noncereal fiber and micronutrient‐rich fruit. The overall composition underlines figs as candidates for dietary interventions targeting glycemic control, cardiovascular disease prevention, and digestive health [23].

**TABLE 1:** Proximate composition of fig fruit paste.

Constituents	Quantity (%)
Moisture	80.16 ± 0.24
Crude protein	1.24 ± 0.03
Crude fat	1.27 ± 0.03
Crude fiber	9.86 ± 0.29
Ash	0.69 ± 0.02
NFE	6.78 ± 0.20

### 3.2. Phytochemical Screening Tests

#### 3.2.1. Total Polyphenol Content (TPC)

Numerous studies have demonstrated the health‐promoting properties of plant‐based bioactive compounds primarily attributed to their strong antioxidant potential [24, 25]. The compositional characteristics of dried figs significantly affect the extraction efficiency of their bioactive constituents, especially polyphenols [26].

The results of fig extracts showed a significant effect (*p* < 0.05) of both solvent type and extraction time on TPC, whereas their interaction was found to be nonsignificant (*p* > 0.05). As shown in Table 2, the highest TPC was obtained in the methanol extract of fig (3.15 ± 0.10 mg GAE/g), followed by water (2.51 ± 0.08 mg GAE/g) and ethanol (2.22 ± 0.08 mg GAE/g) extracts. Extraction time also influenced TPC, with the maximum value (2.81 ± 0.09 mg GAE/g) recorded at 45 min, compared to the lowest (2.51 ± 0.09 mg GAE/g) at 30 min (Table 3). The higher TPC observed in methanol at 45 min may be attributed to enhanced extraction efficiency before the onset of degradation with prolonged exposure. Longer extraction times may enhance solute–solvent interactions; however, longer exposure may lead to thermal or oxidative degradation of polyphenols, explaining the decrease in TPC after 45 min. Similarly, temperature plays a crucial role by increasing solubility and mass transfer rates; moderate heating improves extraction efficiency, whereas high temperatures can cause degradation of heat‐sensitive phenolics [27, 28]. These findings are consistent with those reported by Vinson et al. (2005), who observed similar ranges of TPCs in 1% and 2% fresh and dried fig extracts using methanol and acetone as solvents [29]. Overall, maximizing extraction yield is essential for the effective deployment of fig phenolics in antioxidant‐rich formulations and nutraceutical designs.

**TABLE 2:** Means for total phenolic contents (mg GAE/g) and total flavonoids (mg/g) in fig extracts.

Parameter	Treatments	Time			Means
		30 min	45 min	60 min	
TPC	Methanol	3.03 ± 0.09	3.34 ± 0.11	3.09 ± 0.09	3.15 ± 0.1^a^
	Water	2.41 ± 0.08	2.66 ± 0.09	2.46 ± 0.08	2.51 ± 0.08^b^
	Ethanol	2.09 ± 0.07	2.41 ± 0.07	2.16 ± 0.07	2.22 ± 0.08^c^
	Means	2.51 ± 0.09^b^	2.81 ± 0.09^a^	2.57 ± 0.09^b^	

Flavonoids	Methanol	1.19 ± 0.04	1.30 ± 0.05	1.22 ± 0.05	1.24 ± 0.06^a^
	Water	1.01 ± 0.04	1.11 ± 0.05	1.05 ± 0.04	1.06 ± 0.04^b^
	Ethanol	0.89 ± 0.04	1.05 ± 0.04	0.97 ± 0.05	0.97 ± 0.04^b^
	Means	1.03 ± 0.05^b^	1.16 ± 0.06^a^	1.08 ± 0.05^ab^	

*Note:* Values are expressed as mean ± standard deviation. Means carrying the same letters do not differ significantly (*p* < 0.05). Two‐way ANOVA indicated that solvent type and extraction time significantly affected total polyphenol content (*p* < 0.05), while their interaction was not significant (*p* > 0.05).

**TABLE 3:** Means for DPPH assay (%), *β*‐carotene and linoleic acid assay (%), FRAP assay (μM·TE/g), and ABTS assay (μmol TE/g) of fig extracts.

Parameter	Treatments	Time			Means
		30 min	45 min	60 min	
DPPH assay (%)	Methanol	67.23 ± 2.08	73.74 ± 2.66	69.56 ± 2.64	70.18 ± 2.25^a^
	Water	61.45 ± 2.15	65.27 ± 2.55	62.87 ± 2.20	63.20 ± 2.28^b^
	Ethanol	52.34 ± 1.83	56.25 ± 1.91	54.08 ± 1.84	54.22 ± 1.68^c^
	Means	60.34 ± 2.11^b^	65.09 ± 2.34^a^	62.17 ± 2.36^ab^	

*β*‐Carotene and linoleic acid (%)	Methanol	65.42 ± 3.27	70.74 ± 4.17	64.92 ± 3.51	67.02 ± 3.75^a^
	Water	62.54 ± 3.50	66.98 ± 3.62	58.76 ± 3.17	58.76 ± 3.17^b^
	Ethanol	57.76 ± 3.35	61.86 ± 3.22	54.80 ± 2.85	54.80 ± 2.85^c^
	Means	61.90 ± 2.29^ab^	66.53 ± 3.86^a^	59.49 ± 3.15^b^	

FRAP assay (μM·TE/g)	Methanol	15.59 ± 0.90	17.55 ± 0.95	16.65 ± 0.85	16.59 ± 0.83^a^
	Water	12.78 ± 0.69	14.52 ± 0.78	13.01 ± 0.70	13.44 ± 0.75^ab^
	Ethanol	9.89 ± 0.53	11.16 ± 0.63	10.03 ± 0.54	10.36 ± 0.61^b^
	Means	12.75 ± 0.66^b^	14.41 ± 0.82^a^	13.23 ± 0.75^ab^	

ABTS assay (μmol TE/g)	Methanol	8.55 ± 0.36	10.87 ± 0.53	9.14 ± 0.44	9.52 ± 0.39^a^
	Water	6.96 ± 0.29	8.43 ± 0.39	7.67 ± 0.34	7.69 ± 0.38^ab^
	Ethanol	5.49 ± 0.26	6.89 ± 0.32	6.36 ± 0.27	6.25 ± 0.31^b^
	Means	7.00 ± 0.34^b^	8.73 ± 0.36^a^	7.72 ± 0.31^ab^	

*Note:* Values are expressed as mean ± standard deviation. Means carrying the same letters do not differ significantly (*p* < 0.05).

#### 3.2.2. Flavonoids

Flavonoids are a diverse class of polyphenols, including flavonols, flavones, flavanones, and anthocyanins, which play key roles in scavenging free radicals, chelating metal ions, and modulating cellular signaling pathways. Their presence is strongly associated with antioxidant, anti‐inflammatory, cardioprotective, and anticancer activities. The protective effects of *Ficus carica* (fig) against chronic diseases have been attributed to the antioxidant potential of its flavonoid constituents. In the present study, similar solvent‐ and time‐dependent patterns were observed for flavonoid extraction as those for total polyphenols. Similar solvent‐ and time‐dependent trends were observed for flavonoid extraction. Analysis of variance revealed that the mean squares for total flavonoids differed significantly (*p* < 0.05) with respect to solvent type and extraction time, whereas their interaction effect was not significant (*p* > 0.05). The mean total flavonoid contents (Table 2) obtained using methanol (50% v/v), water, and ethanol (50% v/v) were 1.24 ± 0.06, 1.06 ± 0.04, and 0.97 ± 0.04 mg/g, respectively, with methanol yielding the highest values. Regarding extraction time, the highest flavonoid concentration (1.16 ± 0.05 mg/g) was achieved after 45 min, followed by 60 min (1.08 ± 0.05 mg/g) and 30 min (1.03 ± 0.04 mg/g). These observations are consistent with the literature suggesting methanol’s ability to disrupt plant cellular matrices and enhance flavonoid release compared to less polar solvents. According to previous findings, an increase in methanol concentration enhances the extraction efficiency of flavonoids from fig fruit paste, supporting the present findings that aqueous methanol is a more effective solvent compared to water or ethanol [5]. The antioxidant potentials of various fig varieties have been found with the total flavonoid content to be in the range between 0.96 and 2.01 mg GAE/g fresh weight in methanol extracts. In a study by Kojic et al. (2011), the documented flavonoid content in fig fruit paste was 0.44–2.5 mg/g, and a strong correlation between flavonoids and phenolic content was also found [30]. Given the strong correlation between flavonoid content and radical scavenging potential, these findings afford figs considerable appeal for incorporation into antioxidant‐targeted food formulations and supplements. Their robust antioxidant profile further underscores their relevance to chronic disease prevention.

### 3.3. In Vitro Studies

#### 3.3.1. Free Radical Scavenging Activity (DPPH Assay)

The mean square values for DPPH radical scavenging activity of *Ficus carica* extracts indicated a significant (*p* < 0.05) effect of solvent type and extraction time, whereas their interaction was nonsignificant (*p* > 0.05). As presented in Table 3, antioxidant testing revealed the pronounced efficacy of methanolic extracts, which displayed the highest DPPH radical scavenging capacity (70.18 ± 2.25%), and aqueous (63.20 ± 2.28%) extracts exhibited higher free radical scavenging activities compared to the ethanolic extract (54.22 ± 1.68%). Regarding extraction time, the highest antioxidant activity was observed after 45 min (65.09 ± 2.34%), followed by 60 min (62.17 ± 2.36%) and 30 min (60.34 ± 4.61%), likely due to thermal or oxidative degradation occurring.

Previous studies have reported strong antioxidant activity in fig extracts [31]. For instance, methanolic fig fruit extracts have shown significant DPPH radical scavenging capacity associated with high phenolic content. In addition, the antioxidant activity of fig extracts is influenced by the extraction solvent, as different solvents can extract varying levels of bioactive phenolic compounds [32]. The present data are in accordance with earlier findings, which demonstrated that the methanolic preparation of fig extract exhibited higher antioxidant potential than its ethanolic or aqueous extract under similar conditions. Yang et al. determined the values for DPPH scavenging activity for fig in methanol, ethanol, and water as 92.20 ± 0.52, 91.74 ± 0.47, and 88.36 ± 0.76%, respectively [33]. Furthermore, Faleh et al. estimated DPPH % activity in aqueous fig extracts, and they rendered water a weaker solvent as it extracted less catechin content from fig as compared to methanol [34]. In an analysis, Jahan et al. found that the fruit extract showed 60% DPPH scavenging activity [35]. Among the polyphenols, the compound 3‐O‐(E)‐caffeoyl quinate present in fig has a strong DPPH scavenging activity up to 95.1%. High DPPH scavenging in figs is mechanistically attributable to their rich phenolic matrix, particularly the abundance of caffeoyl quinate, quercetin, and catechin derivatives, which serve as hydrogen donors to stabilize free radicals. Consequently, figs extracted under optimal conditions may contribute to antioxidant functionality in food systems and supplements.

#### 3.3.2. *β*‐Carotene and Linoleic Acid Assay (Antioxidant Activity)

Fig extracts exhibited a significant (*p* < 0.05) difference in antioxidant activity, with significant effects of solvent type and extraction time, but nonsignificant for their interaction effect. Means from Table 3 indicated the highest antioxidant potential (67.02 ± 3.75%) in methanolic extract, followed by aqueous (58.76 ± 3.17) and ethanolic (54.80 ± 2.85%) extracts, respectively. Similarly, time also influenced *β*‐carotene bleaching capacity, which was observed to be maximum (66.53 ± 3.86%) after 45 min and minimum (59.49 ± 3.15%) at 60 min. Current results are in agreement with explorations of previous studies [14, 35]. They noted better antiradical activity of fig fruit methanolic extract than water extract. Martínez‐García et al. determined the antioxidant activity of fig fruit aqueous extract using *β*‐carotene bleaching and lipid peroxidation methods [36]. They used the methanolic extract of fig fruit as a standard and recorded its percent inhibition as 96.03 ± 0.018, suggesting higher antioxidant activity. The antioxidant activity observed here is mechanistically linked to the presence of both phenolics and flavonoids, which neutralize peroxyl radicals formed during oxidation of linoleic acid, thereby protecting the *β*‐carotene substrate. These findings support the feasibility of fig‐derived antioxidants as natural lipid stabilizers in food and nutraceutical formulations.

#### 3.3.3. FRAP Assay

Data regarding mean squares for the FRAP assay showed a significant (*p* < 0.05) effect of both solvent and time on the antioxidant activity of fig extract, while interaction effects were nonsignificant. Means values for the given parameter (Table 3) illustrated that the methanolic extract exhibited maximum reducing power (16.59 ± 0.83 μM·TE/g), while values for water and ethanolic extracts were 13.44 ± 0.75 and 10.36 ± 0.61 μM mg TE/g, respectively. Likewise, a significant difference was noted with respect to the time factor for all extracts, and the highest value (14.41 ± 0.82 μM mg TE/g) was noted after 45 min. FRAP assay revealed the values for methanol and hot water extraction as 126 ± 4.5 and 123 ± 10.8 mg GAE/g, respectively. However, these values did not differ significantly. The results obtained are somewhat comparable to the earlier findings. Ao et al. evaluated the antioxidant capacity of fig extracts at different concentrations [37]. The maximum FRAP value was estimated for 40% methanolic extract after 30 min of extraction. Values for the two extracts obtained through the FRAP assay were determined as 13.4 ± 0.05 and 19.0 ± 0.05 mmol/L Fe^2+^. As the methods used to determine antioxidant activity are highly dependent on the reaction conditions and the reactants, values for the activity are not the same for all methods. In a scientific exploration, a study compared the effectiveness of a developed testing method, rapid electrochemical screening of antioxidant capacity (RESAC), against conventional photometric assay techniques (DPPH and FRAP) and found the techniques highly correlated [38]. Moreover, they recorded FRAP values for three different fig fruit paste samples as 1.146 ± 0.016, 1.053 ± 0.024, and 1.098 ± 0.023 mg TE per mL and found a high correlation (0.9457). The FRAP values indicate strong electron‐donating capacity, a property associated primarily with hydroxyl‐rich phenolic and flavonoid structures prevalent in methanolic extracts. Relevant literature corroborates these findings; methanolic and hot water extractions of figs usually yield high FRAP values, echoing the dominance of phenolic antioxidants in reducing ferric ions. Thus, optimized extraction parameters directly enhance the redox potential of fig extracts, reinforcing their utility in developing naturally sourced antioxidant products.

#### 3.3.4. ABTS Assay

The mean squares for the ABTS assay indicated a significant (*p* < 0.05) effect of both solvent type and extraction time on the antioxidant activity of fig extracts, while their interaction effect was nonsignificant (*p* > 0.05). As presented in Table 3, the methanolic extract exhibited the highest ABTS value (9.52 ± 0.39 μmol·TE/g), followed by the aqueous (7.69 ± 0.38 μmol·TE/g) and ethanolic (6.25 ± 0.31 μmol·TE/g) extracts. With respect to extraction time, the maximum ABTS value (8.73 ± 0.36 μmol·TE/g) was obtained at 45 min, compared to lower values at 30 min (7.00 ± 0.34 μmol·TE/g) and 60 min (7.72 ± 0.31 μmol·TE/g). Comparable findings were reported by a previous study that observed the highest free radical scavenging activity in the methanolic extract of fig fruit paste (9.2 ± 0.1 μg/mL, ABTS assay) [37]. Similarly, Saini et al. reported ABTS cation scavenging activity of methanolic fig fruit extract ranging from 0.27 to 5.57 mg/g [32]. Mechanistically, the high ABTS activity indicates robust hydrogen and electron transfer capabilities among fig‐derived phenolics and flavonoids. As such, figs serve as an exemplary source of multifunctional antioxidants suitable for integration into food matrices to enhance shelf life and health‐promotional properties.

## 4. Conclusion

The dried fig fruit is a valuable source of bioactive compounds with significant antioxidant potential. Methanol proved to be the most effective extraction solvent, yielding the highest total polyphenol and flavonoid contents and antioxidant activity, with optimal results obtained after 45 min of extraction. These findings highlight fig fruit’s potential use in functional and nutraceutical food formulations. However, the study was limited to in vitro antioxidant assays and did not characterize individual phenolic compounds or evaluate bioavailability in vivo. Future research should employ advanced analytical techniques and biological models to better understand the mechanisms and efficacy of fig‐derived antioxidants. Although methanol yielded the highest phenolic recovery, its application in the nutraceutical industry is limited by toxicity and residual solvent concerns. This study utilizes the methanol extract as a reference standard to evaluate the efficiency of Generally Recognized as Safe (GRAS) solvents. Our results highlight that ethanol serves as a potent green alternative, retaining significant bioactivity while ensuring consumer safety. These insights can guide the development of evidence‐based fig‐derived ingredients, beverages, and snacks tailored for athletes, patients, and health‐conscious consumers seeking natural antioxidant support.

**Nomenclature**
GAE: Gallic acid equivalent
DPPH: 2,2‐Diphenyl‐1‐picrylhydrazyl
FRAP: Ferric reducing antioxidant power
ABTS: 2,2′‐Azino‐bis(3‐ethylbenzothiazoline‐6‐sulfonic acid)
NFE: Nitrogen‐free extract
TPC: Total phenolic content
TEAC: Trolox equivalent antioxidant capacity
SD: Standard deviation

## Funding

This research received no external funding.

Open Access publishing was facilitated by the Deanship of Scientific Research (DSR) at King Abdulaziz University, as part of the Wiley–King Abdulaziz University agreement.

## Conflicts of Interest

The authors declare no conflicts of interest.

## Data Availability

The authors declare that the data supporting the findings of this study are available within the paper. Should any raw data be needed in another format, they are available from the corresponding author upon reasonable request.
